# Indentation Size Effect in CoCrFeMnNi HEA Prepared by Various Techniques

**DOI:** 10.3390/ma14237246

**Published:** 2021-11-27

**Authors:** Jaroslav Čech, Petr Haušild, Miroslav Karlík, Jiří Čapek, Filip Průša

**Affiliations:** 1 Faculty of Nuclear Sciences and Physical Engineering, Czech Technical University in Prague, 120 00 Prague, Czech Republic; petr.hausild@fjfi.cvut.cz (P.H.); miroslav.karlik@fjfi.cvut.cz (M.K.); jiri.capek@fjfi.cvut.cz (J.Č.); 2 Department of Metals and Corrosion Engineering, University of Chemistry and Technology, 166 28 Prague, Czech Republic; prusaf@vscht.cz

**Keywords:** CoCrFeMnNi, HEA, nanoindentation, indentation size effect, microstructure

## Abstract

High entropy alloys (HEAs) are materials of great application potential and which have been extensively studied during the last two decades. As the number of possible element combinations is enormous, model materials representing certain groups of HEAs are used for the description of microstructure, properties, and deformation mechanisms. In this study, the microstructure and mechanical properties of the so-called Cantor alloy composed of Co, Cr, Fe, Mn, and Ni in equiatomic ratios prepared by various techniques (casting, melt-spinning, spark plasma sintering) were examined. The research focused on the indentation measurements, namely, the indentation size effect describing the evolution of the hardness with penetration depth. It was found that the standard Nix–Gao model can be used for this type of alloy at higher penetration depths and its parameters correlate well with microstructural observations. The Nix–Gao model deviates from the measured data at the submicrometer range and the applied modification affords additional information on the deformation mechanism.

## 1. Introduction

High entropy alloys (HEAs) are defined as alloys composed of at least five principal elements in equiatomic or near-equiatomic concentrations (ranging from 5 to 35%). The multiprincipal element character of HEAs, firstly described in [1,2], results in so-called core effects, which include the high-entropy effect, sluggish-diffusion effect, severe lattice-distortion effect, and cocktail effect. These effects, described in detail, for example, in [3], are responsible for the interesting properties of the HEAs, giving them great application potential. As an example, we can state high strength, hardness, wear resistance, high-temperature strength, creep resistance, structural stability, good corrosion, and oxidation resistance [3]. Their applications are not limited to structural applications only; they can also be used as hydrogen storage materials, radiation-resistant materials, diffusion barriers, and soft magnetic or biocompatible materials [3].

One of the main characteristics of these alloys, which is a consequence of a high mixing entropy, is the formation of a single-phase solid solution. The most-commonly studied groups of HEAs are based on face-centered cubic (fcc) and body-centered cubic (bcc) phases. Many transition states with mixed structures can be obtained, which can lead to other new materials with excellent properties. As the number of possible element combinations is enormous, knowledge of them is still limited, and therefore, further research is needed. For this reason, a model material representing a certain group of HEAs is usually used for the characterization of their physical and mechanical properties. One of the most-studied materials is the so-called Cantor alloy CoCrFeMnNi [1] with fcc structure.

This alloy can be prepared by various techniques, resulting in different microstructures offering various mechanical properties. In addition to traditional vacuum arc melting (with subsequent thermo-mechanical processing), melt-spinning (MS) or mechanical alloying (MA), with subsequent spark plasma sintering (SPS), can be used. During the melt-spinning process, molten metal is ejected onto the surface of the quickly rotating wheel, which results in the fast cooling of the material in the form of ribbons. This method leads to a very fine-grained microstructure and, in the case of certain alloys, even to the formation of bulk metallic glasses. Various combinations of parameters, such as the wheel’s rotation rate and its cooling, gas ejection pressure, and/or melt temperature, affect the resulting dimensions of the ribbons, their microstructure [4,5,6], and their mechanical or magnetic properties [7,8].

During mechanical alloying, a mixture of pure element powders is placed into the ball mill, where they undergo repeated cold welding, fracturing, and rewelding, until they form a solid solution with a homogenous and very fine-grained structure with a significant amount of imposed plastic deformation [9,10]. The following powder compactization is frequently processed by the method of spark plasma sintering, where an electric current passes through the pressed powder which is heated by the Joule effect. As the whole process is very fast, it prevents grain coarsening and results in a sintered sample with superior properties [11,12,13].

Properties of the Cantor alloy as a model material have already been extensively studied. Studies on diffusion [14], microstructure, and texture after cold rolling and annealing [15], grain growth kinetics and Hall–Petch relationship [16,17], and the effect of temperature and grain size on the tensile properties and deformation mechanisms [18] have been reported. Nanoindentation creep and the effect of the loading rate for Cantor alloy processed by high-pressure torsion were investigated in [19]. The effects of initial powder state, milling time, sintering temperature, and contamination on the microstructural evolution and mechanical properties of SPS-processed CoCrFeMnNi HEAs were investigated, for example, in [20,21,22,23].

On the other hand, not much attention was focused on the indentation size effect (ISE) in HEAs. ISE is frequently observed in the nanoindentation of metals and alloys, and it is manifested by an increase of hardness with a decreasing penetration depth. The explanations of this phenomenon are based on the concepts of strain gradient plasticity. In the dimensions where the density of statistically stored dislocations (SSDs) is too low to accommodate the shape of the indenter (i.e., in low penetration depths), geometrically necessary dislocations (GNDs) must be considered. The classical Nix–Gao model [24] is valid for higher indentation depths, but it fails in the submicrometer range. The overestimation of hardness at low depths by this model is believed to be caused by the strong repulsive force between GNDs [25]. Various models taking into account the spread of the GNDs out of the supposed volume by its extension, limiting the maximum density of GNDs, or their non-uniform distribution, were proposed [26,27,28,29,30,31].

In the presented study, we focused on ISE in HEA in various microstructural and deformation states. For this reason, the model CoCrFeMnNi high-entropy alloy was prepared by various techniques, resulting in different grain sizes and dislocation densities. The main aim was to correlate the observed microstructure with nanoindentation measurements, namely, the effect of structural unit size on the indentation size effect.

## 2. Materials and Methods

### 2.1. Materials Preparation

All the samples were composed of Co, Cr, Fe, Mn, and Ni elements in equiatomic (i.e., 20 at. %) portions. The cast sample was prepared by induction melting in a protective argon atmosphere at a temperature of 1800 °C. The melt was then homogenized for several minutes by eddy currents. An ingot with a diameter of 14 mm and a total length of 150 mm was cast into a steel crucible.

To prepare melt-spun samples, the material was placed into an induction melting furnace, which was evacuated and filled with a protective Ar atmosphere (10^3^ Pa). Thin ribbons of an average thickness of 30 µm and width of 2–5 mm were formed by ejecting the molten alloy, using an Ar overpressure of 3 × 10^4^ Pa, to the water-cooled Cu wheel rotating at a circumferential speed of 30 m/s. Two types of melt-spun ribbons were prepared by different cooling methods at the end of the process. After the separation from the cooling wheel, the ribbons were cast into water (samples denoted MS-water) to maximize the cooling effect of the process, or were cooled down in the ambient air (samples denoted MS-air).

Pure elements powders (Sigma Aldrich, Saint Louis, MO, USA) with purity ≥99.9% (Co), ≥99.7% (Ni), and ≥99% (Cr, Fe, Mn) were blended and mechanically alloyed in a planetary ball mill (Retsch PM-100, Haan, Germany). Overall, 20 g of the powders was mixed to form equiatomic composition (4.20 g of Co, 3.71 g of Cr, 3.98 g of Fe, 3.92 g of Mn, and 4.19 g of Ni). The ball-to-powder ratio was 15:1. The mechanical alloying was carried out in a vacuum for a total duration of 24 h at a rotational speed of 400 rpm. The vial of the mill and milling balls were made of AISI 420 stainless steel (nominal composition in wt.%: C > 0.15, Mn < 1, Si < 1, P < 0.04 S < 0.03, Cr: 12.0–14.0, Fe: bal.). The fully homogenized powder was compacted by a SPS machine HP D10 device (FCT Systeme GmbH, Rauenstein, Germany). The disc, with a diameter of 20 mm, was sintered at a temperature of 1100 °C using a pressure of 48 MPa. The heating and cooling rate was 100 °C/min and the dwell time at the maximum temperature was 5 min.

### 2.2. Characterization Techniques

Metallographic cuts were prepared by the standard procedures of grinding on abrasive papers and polishing with diamond pastes, and finished by polishing with 0.04 µm colloidal silica. The microstructure of the prepared samples was revealed by etching in a solution of HCl, HNO_3_, and water in the ratio 1:1:1. The microstructure was observed in the backscattered electron (BSE) signal with a JEOL JSM IT500HR (JEOL, Tokyo, Japan) scanning electron microscope (SEM). The chemical composition was analyzed with an adjacent energy-dispersive spectrometer JED-2300. The grain size was evaluated by the linear intersection method.

The phase composition was studied with a PANalytical X’Pert Pro X-Ray diffractometer (PANalytical, Almelo, The Netherlands) in Bragg–Brentano geometry with Co radiation.

Nanoindentation tests for the instrumented hardness (*H*) and Young’s modulus (*E*) measurements were carried out on an NHT^2^ nanoindentation tester (Anton Paar, Graz, Austria) equipped with a Berkovich diamond indenter tip. Measurements with partial unloadings (continuous-multi-cycle—CMC) were performed in order to study the mechanical properties of the samples with increasing penetration depths. The loading cycle consisted of 30 loading–unloading phases, with the maximum load ranging from 0.1 mN to 500 mN for cast and SPS samples. The loading, holding period, and unloading of every cycle lasted 10 s, 5 s, and 10 s, respectively. Indentation tests on melt-spun samples were performed on the longitudinal cross-section of the ribbons. In order to avoid the effect of the surrounding material on the measured values of the mechanical properties for melt-spun samples, tests consisted only of 20 cycles with a maximum load of 100 mN. Unloading parts of the measured load–depth curves were evaluated by the Oliver–Pharr method [32,33], and the instrumented hardness and Young´s modulus were calculated according to standard ISO 14577 [34].

To avoid the hardening effect and/or to release the residual stresses which can be imposed into the surface layers during grinding and polishing, the surface of the samples was finished by long-term polishing in colloidal silica suspension. The measured CMC indentation load–depth curves were compared with standard measurements (30 s loading, 10 s holding period, 30 s unloading) to confirm that thermal drift did not affect the measured results.

For the evaluation of instrumented hardness evolution with the depth, the Nix–Gao model [24], represented by Equation (1), was applied for the data at depths higher than 1 µm:

(1) $$H=H_{0}\sqrt{1+\frac{h*}{h_{c}}}$$

where *H* is the instrumented hardness for a given contact penetration depth *h_c_*, *H*_0_ is the hardness in the limit of infinite depth, and h* is a characteristic length parameter. As the Nix–Gao model becomes invalid at the submicrometer range, a modification that assumes that the number of dislocations contained in the effective plastic zone under the contact area incrementally scales with the penetration depth with *h^m^* [30] was used for the description of *H*-*h_c_* dependency at lower depths, according to Equation (2):

(2) $$H=H_{0}\sqrt{1+\frac{h*}{h}\left(1-e^{-\frac{h^{n}}{h_{1}}}\right)}$$

where *h*_1_ and *n* are fitting parameters. The hardness values obtained for the lowest applied loads were excluded from the analysis, as they could be significantly affected by an incompletely developed plastic zone around the indent.

## 3. Results and Discussion

### 3.1. Structural Characterization

**Figure 1:**
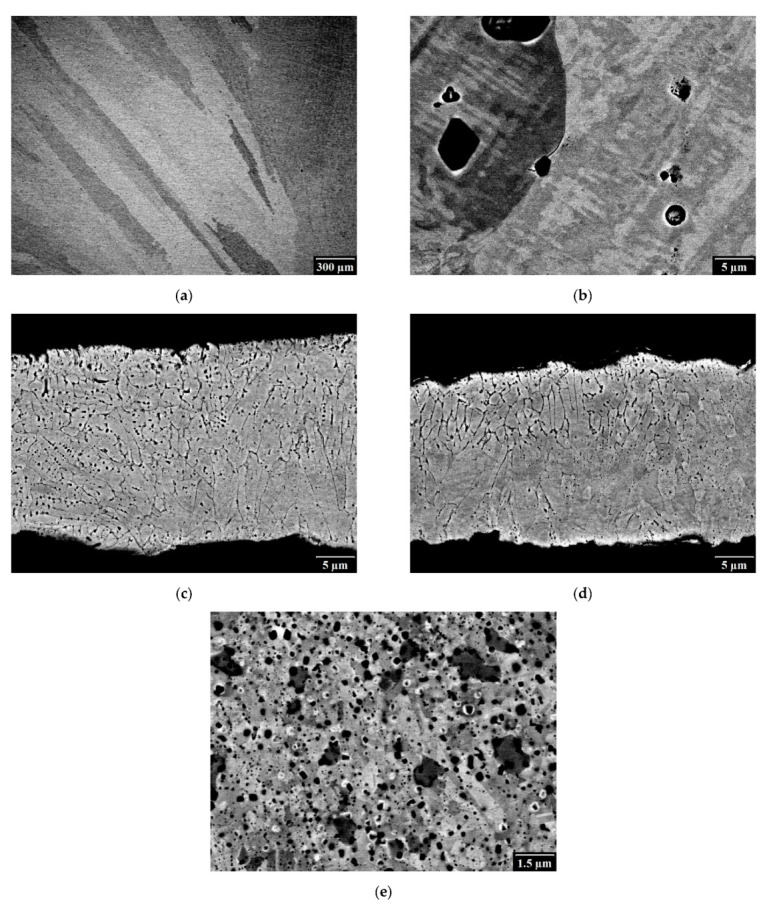
Microstructure of CoCrFeMnNi samples: (**a**,**b**) cast; (**c**) MS-water; (**d**) MS-air; (**e**) SPS.

The microstructure of CoCrFeMnNi samples is shown in Figure 1. The cast sample (Figure 1a) shows a typical casting structure with large grains (in the order of hundreds of µm up to a few mm) elongated in the radial direction of the ingot. Higher magnifications (Figure 1b) show a substructure in the form of cells with the size of a few µm. The homogeneity of the prepared cast sample was sufficient, as has been proven by our previous research, which prepared the same alloy using the same or a similar set of parameters [21]. Moreover, considering the melting points of each individual element, chromium exhibited the highest one, making it alone impossible to melt at the temperature of 1800 °C. This major setback is, however, balanced by its ability to dissolve in molten Co, Mn, Ni, etc., at significantly lower temperatures, as is shown, for example, in the binary phase diagrams for Co-Cr, Mn-Cr, Ni-Cr, and others. Considering that Co, Ni, Mn, and Fe are molten at this temperature, the overall Cr atomic fraction decreases (compared to the theoretical 50 at. % for the binary system of X-Cr alloys) further decreasing the melting temperature of X-Cr for such a complex system. This ability is often used for a wide range of metallic materials with high melting temperatures, which would not otherwise melt at a given temperature, but that are able to dissolve in molten material at a much lower temperature. This presumption is also confirmed by the DTA analysis of CoCrFeNiMn materials, which have a melting temperature of around 1250 °C [21], a temperature far below the temperature used (1800 °C). The homogeneity of the sample was verified by XRF measurements at different areas of the ingot, and no significant differences were found.

The thickness of the melt-spun ribbons varied between 15 and 60 µm, with the average thickness around 30 µm. The droplets of the material were occasionally observed. The solidification started on the side adjacent to the cooling wheel (bottom side of Figure 1c,d) and the grains had a columnar shape. The grains were significantly smaller than in the case of the cast sample, which is characteristic of the melt-spinning process. Their size, measured in the direction of the ribbon axis, was about 1.1 µm for both melt-spun samples.

**Figure 2:**
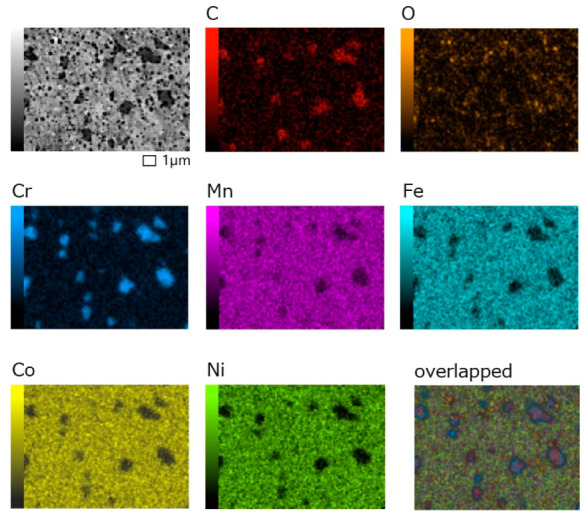
Elemental map of the SPS sample.

The microstructure of the SPS sample (Figure 1e) was the finest, with an average fcc grain size of 0.5 µm and negligible porosity. The grains were equiaxed. The small grain size is typical for the samples prepared by MA and SPS, as the compaction process is very fast and, therefore, the grain coarsening is avoided. The elemental map of the SPS sample (Figure 2) shows that, except for the primary elements, carbon and oxygen were present. It can be seen that C is usually coupled with Cr, which shows the formation of chromium carbides (larger dark particles in Figure 1e), which is typical for SPS samples contaminated by C near the surface of the SPS graphite mold [20]. Oxygen is present in the form of oxides, which were evenly distributed in the structure (small black particles in Figure 1e).

The results of the chemical composition measurements are presented in Table 1. In addition to the primary elements, impurities in the form of Si, O, and Al are present. Their amount is negligible, except for the oxygen in the SPS sample. Oxygen is introduced into the powder during the ball-milling process, and its presence cannot be fully avoided, even if the milling is performed under vacuum or in a protective atmosphere [35].

**Figure 3:**
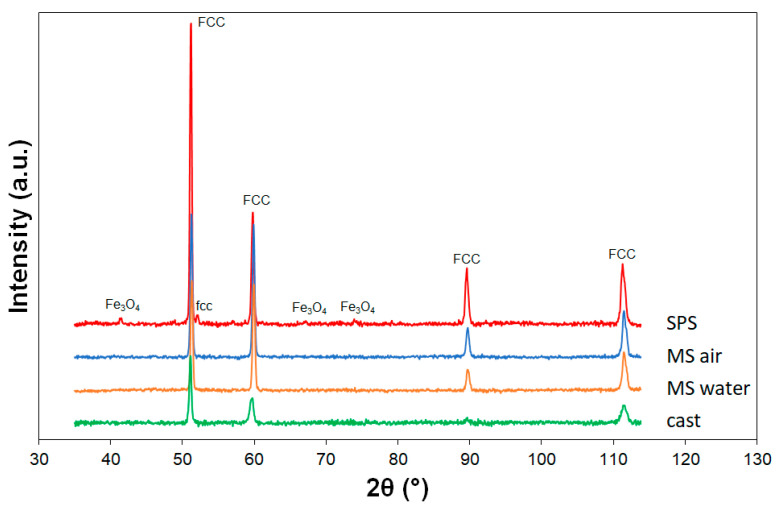
XRD diffraction patterns of CoCrFeMnNi samples.

The results of X-ray diffraction in Figure 3 show that all the samples are formed by a single-phase solid solution with a face-centered cubic structure with lattice parameter *a* = 3.597 A. The diffraction pattern of the SPS sample shows a small peak of the second fcc phase (lattice parameter *a* = 3.537 A) and peaks corresponding to the structure of iron oxide Fe_3_O_4_, which confirms the microstructural and chemical observations.

### 3.2. Indentation

**Figure 4:**
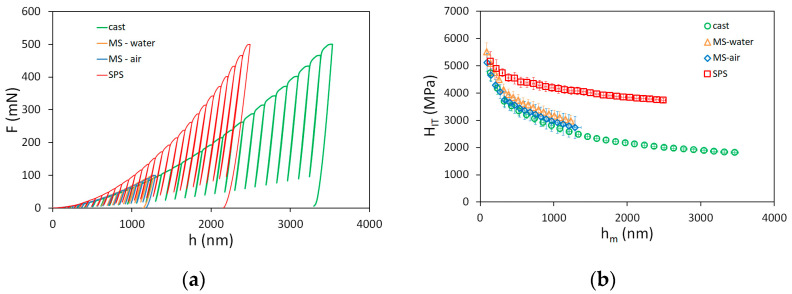
(**a**) Indentation force–depth curves; (**b**) hardness as a function of indentation depth of CoCrFeMnNi samples.

Typical indentation force–depth curves with partial unloadings are shown in Figure 4a. The maximum penetration depth for the cast sample was about 3.5 µm, and about 2.5 µm for the SPS sample. As the thickness of the melt-spun ribbons was limited, the maximum penetration depth for the MS samples was kept at approximately 1.3 µm. The correctness of the measurements was confirmed by the measured values of Young´s modulus, which were constant through the whole range of analyzed depths. The values reached approximately 200 GPa and are summarized in Table 2. Slightly higher values obtained for the SPS sample are probably a consequence of impurities present in the form of carbides and oxides.

The evolution of instrumented hardness with indentation depth is shown in Figure 4b. It can be seen that the hardness decreases with the depth, which is the result of the indentation size effect. At low penetration depths, the hardness is nearly similar for all samples, as it is governed by the density of geometrically necessary dislocations. The microstructure of the samples becomes more important for the hardness values at higher penetration depths. Hardness is the highest for the SPS sample, as the grain size is the smallest. It could be also supposed that the dislocation density is the highest for the SPS sample, which is the consequence of the mechanical alloying imposing high deformations into the material. The melt-spun samples are nearly similar (the sample finished in water was slightly harder) and softer than the SPS sample. This is the result of the larger grain size and smaller dislocation density, as the samples are not supposed to be deformed during the preparation process. The lowest hardness was observed for the cast sample, with large grains and no imposed plastic deformation during the casting process.

**Figure 5:**
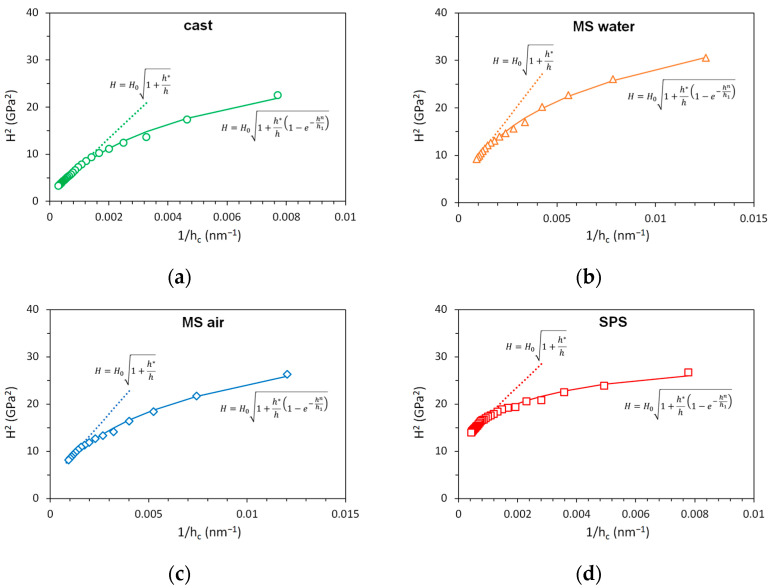
Depth dependence of hardness of CoCrFeMnNi samples: (**a**) cast; (**b**) MS-water; (**c**) MS-air; (**d**) SPS.

**Figure 6:**
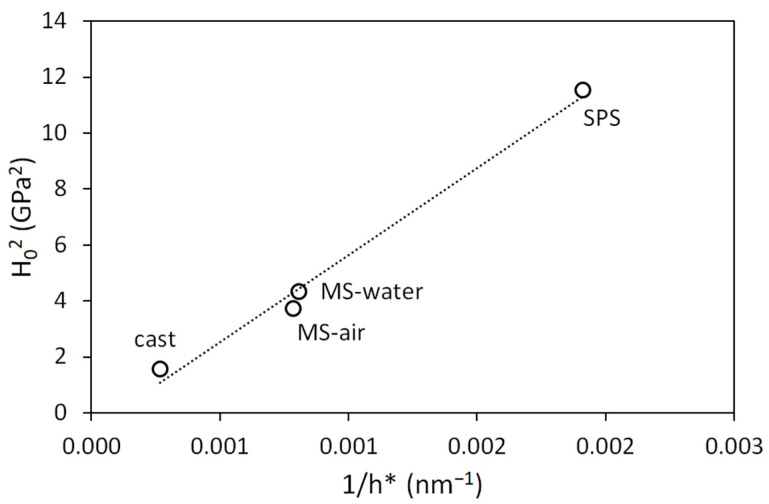
*H*_0_^2^ vs. 1/h* dependency of CoCrFeMnNi samples.

Figure 5 shows the depth dependence of instrumented hardness in the form of *H*^2^ vs. 1/*h* plots. The Nix–Gao model (dotted line) according to Equation (1) fits the data well for depths higher than 1 µm. The fitting parameters are summarized in Table 2. Hardness in the limit of infinite depth *H*_0_ goes to 3.4 GPa for the SPS sample, 2 GPa for melt-spun samples, and 1.3 GPa for the cast sample. It corresponds well to the values shown in Figure 4b. The values of characteristic length h* decrease with increasing *H*_0_. Figure 6 shows the linear relation between *H*_0_^2^ and 1/h*, which confirms the validity of the Nix–Gao model. The slope of this dependency is given by the indenter geometry, the Burgers vector, and the material shear modulus. The value of *H*_0_ depends on the microstructure of the material, namely, the density of statistically stored dislocations. The characteristic length h* obtained from the Nix–Gao fit shows a very good correlation with the observed size of the structural unit, as can be seen in Table 2. For the melt-spun samples, the grain size is measured in the direction of the ribbon axis (where the grain size is the lowest). This direction is crucial, since the distance to the nearest obstacle is important for the propagation and pile-up of dislocations. For the cast sample, the governing dimension seems to be the size of the substructural unit (cell or subgrain), as the (high-angle) grain boundaries are too distant to have any influence on the ISE. Based on these observations, it can be concluded that the proper analysis of indentation data and indentation size effect can give information about the structural parameters governing the deformation properties of the material. This information cannot be directly obtained, e.g., by microstructural observations.

**Figure 7:**
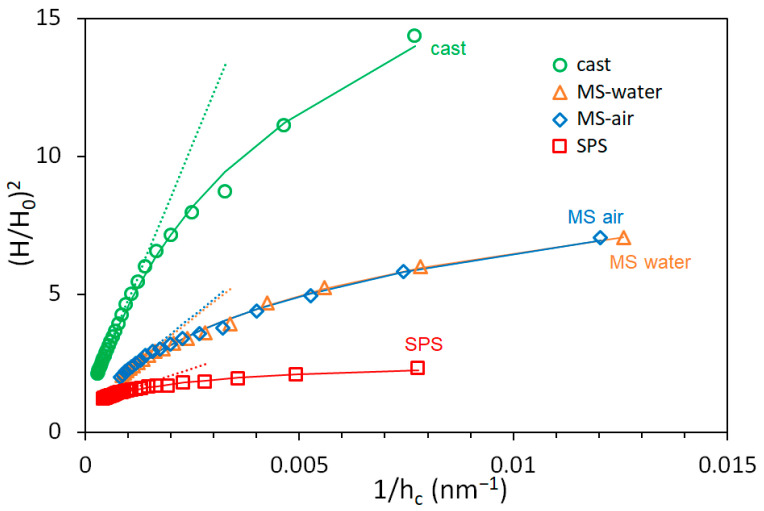
Normalized *H*^2^ vs. 1/*h* plot of CoCrFeMnNi samples.

The normalized *H*^2^ vs. 1/*h* plot for all four samples is shown in Figure 7. It documents that the size effect is the most pronounced for the cast sample and less pronounced for the SPS sample. This is given by the density of the statistically stored dislocations. As the overall indentation response is given by the sum of geometrically necessary and statistically stored dislocations, fewer GNDs are necessary to accommodate the deformation caused by the indenter at low depths in the samples with a higher density of SSDs. The density of the SSDs is highest in the SPS sample; thus, the indentation size effect is less pronounced. On the other hand, the lowest density of the SSDs in the cast sample results in the most distinctive size effect.

**Figure 8:**
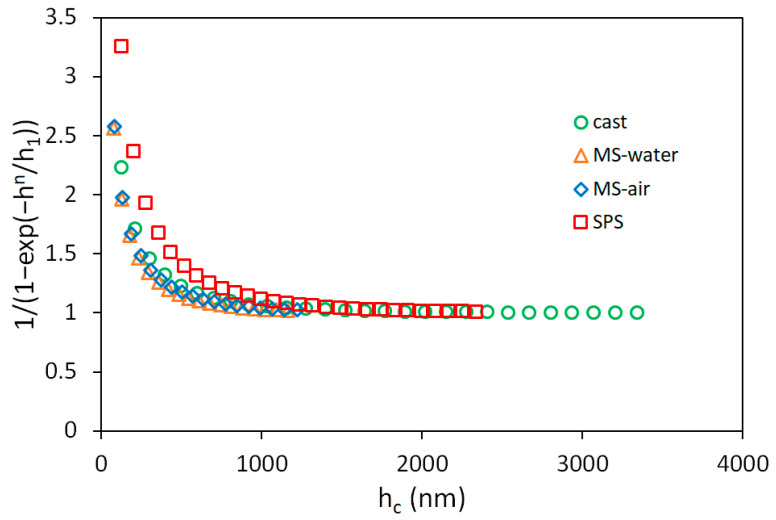
Expansion of effective plastic zone volume of CoCrFeMnNi samples.

The Nix–Gao fit of instrumented hardness data is not valid at depths lower than approximately 1 µm (Figure 5 and Figure 7). The hardness data at these depths are well described by the model represented by Equation (2). The fitting parameters *h*_1_ and *n* are summarized in Table 2. These parameters are nearly similar for cast and melt-spun samples, and slightly higher for the SPS sample. Higher parameters cause the hardness data to start to deviate from the Nix–Gao model sooner (at higher penetration depths). The expansion of the effective plastic zone under the indenter that causes the deviations from the Nix–Gao model is shown in Figure 8. The difference between the SPS sample and the other samples could probably be caused by the presence of impurities in the SPS sample coming from the preparation process. Slightly different chemical compositions can also change the lattice friction stress, which affects the onset of the deviation from the Nix–Gao model [30]. Small oxide particles can act as local stress concentrators and obstacles for dislocation movement, and in this case, other deformation mechanisms, e.g., twinning, can start to play a role [36]. A yet-deeper understanding of this phenomenon is needed.

Overall, it was shown that different grain sizes, the density of SSDs, and the presence of impurities, which are the consequence of the preparation method, have a crucial effect on the performance of CoCrFeMnNi alloy at the nano/micro scale. As the size of some samples is limited (i.e., melt-spun ribbons in this study), classical macroscopic tests (e.g., tensile tests) cannot be performed and the nanoindentation testing has an irreplaceable role in their examination. Moreover, nanoindentation testing and the analysis of the indentation size effect, compared to other methods which can be used for the investigation of the mechanical behavior at the nano/micro scale (e.g., micropillar compression, microcantilever bending [37,38]) is relatively easy to perform and, thus, well-suited for the description of the deformation phenomena appearing at the very local scale.

## 4. Conclusions

A high-entropy alloy with CoCrFeMnNi equiatomic composition was investigated in this study. Microstructure and mechanical response to indentation measurements were examined for the samples prepared by casting, melt-spinning, and mechanical alloying, with subsequent spark plasma sintering. Grain size was the lowest (submicrometer range) for the mechanically alloyed sample compacted by SPS. Melt-spun samples have micrometric grains with the orientation locally affected by directional solidification. Casted sample shows very large grains; nevertheless, the performance of this alloy is governed by the size of the substructural units, which are only slightly larger than the grains of the melt-spun samples. The results show that the preparation method, and thus the microstructure, significantly affect the hardness of the material and its response to the deformation imposed by the penetrating indenter. The indentation size effect in the studied alloy, which is usually used as a model material for HEAs with fcc structure, are well-described by the Nix–Gao model at depths higher than 1 µm, and by a modified Nix–Gao model that takes into account the repulsive forces between dislocations at lower penetration depths. The results show a very good correlation between the characteristic length parameters obtained from the indentation data and the sizes of the observed microstructural features in differently prepared alloys. It was found that the sample with the lowest hardness and lowest dislocation density exhibits the most pronounced indentation size effect, and vice versa.

## Figures and Tables

**Table 1:** Analyzed chemical composition of CoCrFeMnNi samples (at. %).

	Co	Cr	Fe	Mn	Ni	Si	O	Al
cast	22.4 ± 0.2	15.0 ± 0.2	22.1 ± 0.2	20.3 ± 0.2	19.5 ± 0.2		0.7 ± 0.1	
MS-water	20.4 ± 0.2	19.9 ± 0.2	20.6 ± 0.2	18.3 ± 0.2	18.7 ± 0.2	0.8 ± 0.1	1.3 ± 0.1	
MS-air	20.0 ± 0.2	20.0 ± 0.2	20.4 ± 0.2	19.5 ± 0.2	18.5 ± 0.2	0.2 ± 0.1	1.4 ± 0.1	
SPS	18.4 ± 0.1	19.3 ± 0.1	20.3 ± 0.1	18.3 ± 0.1	17.9 ± 0.1	0.1 ± 0.03	5.5 ± 0.1	0.2 ± 0.03

**Table 2:** Parameters of ISE models (*H*_0_, h*, *h*_1_, *n*), Young´s modulus (*E*), and structural unit size.

	*H*_0_ (MPa)	h* (nm)	*h*_1_ (nm)	*n* (-)	*E* (GPa)	Structural Unit Size (nm)	Structural Unit
cast	1252	3761	73	0.78	204.4	4908 ± 542	cell or subgrain
MS-water	2080	1239	60	0.78	193.1	1083 ± 88	grain (direction of ribbon axis)
MS-air	1931	1274	57	0.75	194.8	1078 ± 141	grain (direction of ribbon axis)
SPS	3399	523	201	0.89	218.8	502 ± 38	grain

## Data Availability

Data available upon request from the corresponding author.
